# Structured template for standardised reporting of non-contrast computed tomography in urinary stone disease as a superior tool for urologists – a randomised controlled trial

**DOI:** 10.1186/s12894-026-02134-0

**Published:** 2026-04-06

**Authors:** Maximilian Glienke, Maximilian von Bargen, Konrad Wilhelm, Arkadiusz Miernik, Elmar Kotter, Jakob Neubauer, Jakob Weiß, Philipp Arnold, Christian Gratzke, Martin Schoenthaler

**Affiliations:** 1 Department of Urology, Faculty of Medicine, Medical Centre - University of Freiburg, Hugstetter Str. 55, Freiburg, 79106 Germany; 2 Department of Radiology, Faculty of Medicine, Medical Centre - University of Freiburg, Hugstetter Straße 55, Freiburg, 79106 Germany

**Keywords:** Structured reporting, Computed tomography (CT), Urinary stones

## Abstract

**Background:**

Standardised structured reporting (SR) aims to improve the clarity, completeness, and clinical applicability of radiological reports. For non-contrast computed tomography (NCCT) of urinary stones, a reporting template was developed collaboratively by the German Radiologic Society and the German Society of Urology. This study evaluates urologists’ satisfaction with SR compared to free-text reports (NR).

**Methods:**

A randomised controlled trial was conducted with 200 NCCT reports (100 SR, 100 NR). Five urologists of varying experience levels assessed the reports using a questionnaire, covering content, formal aspects, clinical consequences, and overall report quality. Scores were based on a 6-point Likert scale (0–6).

**Results:**

Structured reports significantly outperformed free-text reports in all evaluated domains (*p* < 0.001). SR provided clearer descriptions of stone characteristics and radiographic signs, enabling precise clinical decision-making. The overall satisfaction score for SR was 5.83 ± 0.56 compared to 4.18 ± 1.13 for NR. Formal aspects, such as report structure, also showed marked improvements in SR.

**Conclusion:**

The findings demonstrate SR’s superiority in clinical utility, completeness, and clarity. By facilitating Artificial Intelligence integration and secondary data analysis, SR offers additional advantages. Broader adoption requires promoting user acceptance and validating translated versions for international use.

**Supplementary Information:**

The online version contains supplementary material available at 10.1186/s12894-026-02134-0.

## Background

There is broad consensus among radiological societies regarding the need for improving the structure and standardisation of radiological reports [1, 2]. The primary goals are to harmonise the terminology used and improve the quality of reporting in terms of completeness, avoiding redundancies or ambiguities, and enhanced clarity and readability [3]. These factors have a potential improve clinical applicability and ultimately treatment planning. Moreover, structured reporting (SR) facilitates the comparability of findings over time and later artificial intelligence (AI) processing.

The German Radiologic Society (*Deutsche Röntgengesellschaft*, DRG) together with societies of various medical fields have developed several templates for the standardised reporting (SR) of various imaging modalities in conjunction with different diseases. The templates are available on the DRG website (www.befundung.drg.de). In close cooperation with the German Society of Urology (DGU) and the DRG, our group has developed the first template for SR of non-contrast enhanced computed tomography (NCCT) for urinary stones [3]. The template is available free of charge on the DRG website (https://www.befundung.drg.de/de-DE/3199/befundvorlagen/041807.2.2203092150-ct_urolithiasis.html/).

The widespread use of such a template requires its future users to accept it, namely both radiologists and the referring physicians. The present study evaluates urologists’ satisfaction comparing free-text reports and reports relying on the DGU/DRG template in a randomised controlled trial.

## Materials and methods

**Fig. 1:**
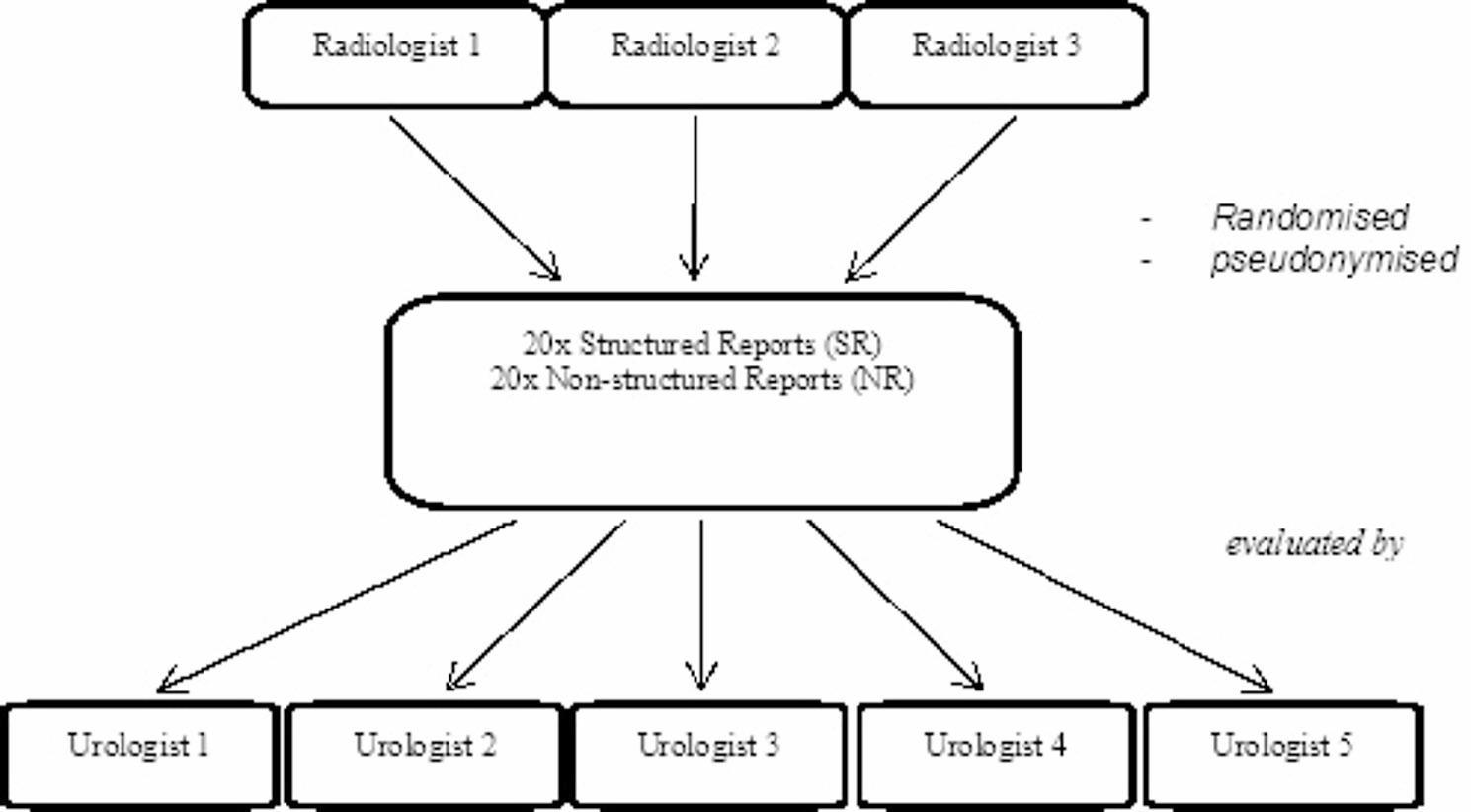
Study Design. Three radiologists created 20 structured and 20 non-structured reports. Reports were pseudonymised and evaluated by five urologists

Our study included 200 evaluations made by five urologists at different training levels - two urologists with two years and three urologists with at least 10 years of experience in endourology. The urologists evaluated 40 radiology reports of NCCTs each, which had been created by three radiologists for the first consecutive patients, between September 2022 and December 2022, who underwent NCCT in our emergency department with a clinical suspicion of urinary stone disease. A computer-generated random allocation sequence was created using simple randomisation (1:1 ratio) without restrictions. Based on this sequence, each of the three radiologists (with three to five years of experience) was assigned to write reports on NCCTs describing stones of the upper urinary tract using either the template or a free-text format. The reports were pseudonymised, reformatted in a uniform fashion, and presented to the urologists in random sequence (Fig. 1). This randomised study was conducted as a single-centre trial at the Department of Urology and Radiology, University Medical Centre Freiburg, Germany. This study was conducted and reported in accordance with the CONSORT 2025 guidelines for randomized controlled trials.

To evaluate the urologists’ acceptance and satisfaction with the template, we used a modified questionnaire as suggested by Sobez et al. [4]. We translated (German) and modified the questionnaire to adapt to the topic of urinary stones. It comprises the four domains content, formal aspects, clinical consequences, and overall report quality, including 1–4 items each. Items had to be rated by the urologists applying a 6-point Likert scale (0="strongly disagree” to 6="strongly agree”) (Supplementary Table S1).

The template was developed following the “process of development, publication and continued updating of report templates” as proposed by the DRG [5]. The template comprises six organ domains – e.g., “right kidney”, and 21 items to be addressed by inserting numerical quantity values or using drop-down menus [3].

We performed a power analysis to estimate the number of reports needed in each group. The endpoint for power analysis was to identify significant differences in the report’s quality aspects between groups. To achieve a power level of 0.80 with a 0.05 significance level, of we needed 20 patients with five reports each in both groups. This study is registered in the German Clinical Trials Register (DRKS) under the registration number DRKS00031813.

### Statistical analysis

The Mann-Whitney U-test was used to assess differences in Likerts‘ response categories. Difference in age, BMI, and stone size were calculated with a t-Test for two independent samples in all randomised patients. The chi-quadrat test was used to compare the presence of ureteral and kidney stones. The probability level of *p* < 0.05 was considered statistically significant for all tests. Adequate statistical methods were applied using Python-based software tools (Python version 3.10.10).

## Results

This study is based on 20 structured reports (SR) and 20 non-structured reports (NR). Basic patient and stone characteristics did not differ significantly (Table 1). The mean patient age in the SR cohort was 51.35 ± 16.51 years (range 22–77) versus 50.95 ± 20.13 (range, 18–85) in the NR cohort (*p* = 0.94). The mean BMI in the SR group cohort was 28.59 ± 5.68 (range 20.2–46.1) versus 26.65 ± 4.92 (range 17.9–38.8) in the NR cohort (*p* = 0.11). A urinary stone was described in all reports. Average stone size was 7.35 ± 6.9 mm in the SR cohort and 6.3 ± 3.2 mm in the NR cohort, respectively (*p* = 0.54). Ureteral and kidney stones were equally distributed between the two groups (*p* = 0.77).

**Table 1:** Baseline characteristics of patient cohort. SR = structured reports, NR = non-structured reports. SD = Standard Deviation

	SR	SD	NR	SD	*p*
Age (y)	51 (22–77)	16.51	50.95 (18–85)	20.13	0.94
BMI (kg/m2)	28.59 (20.2–46.1)	5.68	26.65 (17.9–38.8)	4.92	0.11
Stone size (mm)	7.35 (2–34)	6.9	6.3 (2–13)	3.2	0.54
Kidney stones	6/20		5/20		1
Ureteral stones	14/20		15/20		

Assessments in the SR yielded significantly higher average scores than the unstructured free-text reports. This was true for all domains and each item in the questionnaire (*p* < 0.001). These results applied also to each of the five urologists independent of their experience. Urologists assigned the SR a mean score of 5.66 ± 0.72 compared to 3.89 ± 1.28 in unstructured reports (*p* < 0.001) (Supplementary Table S2).

**Fig. 2:**
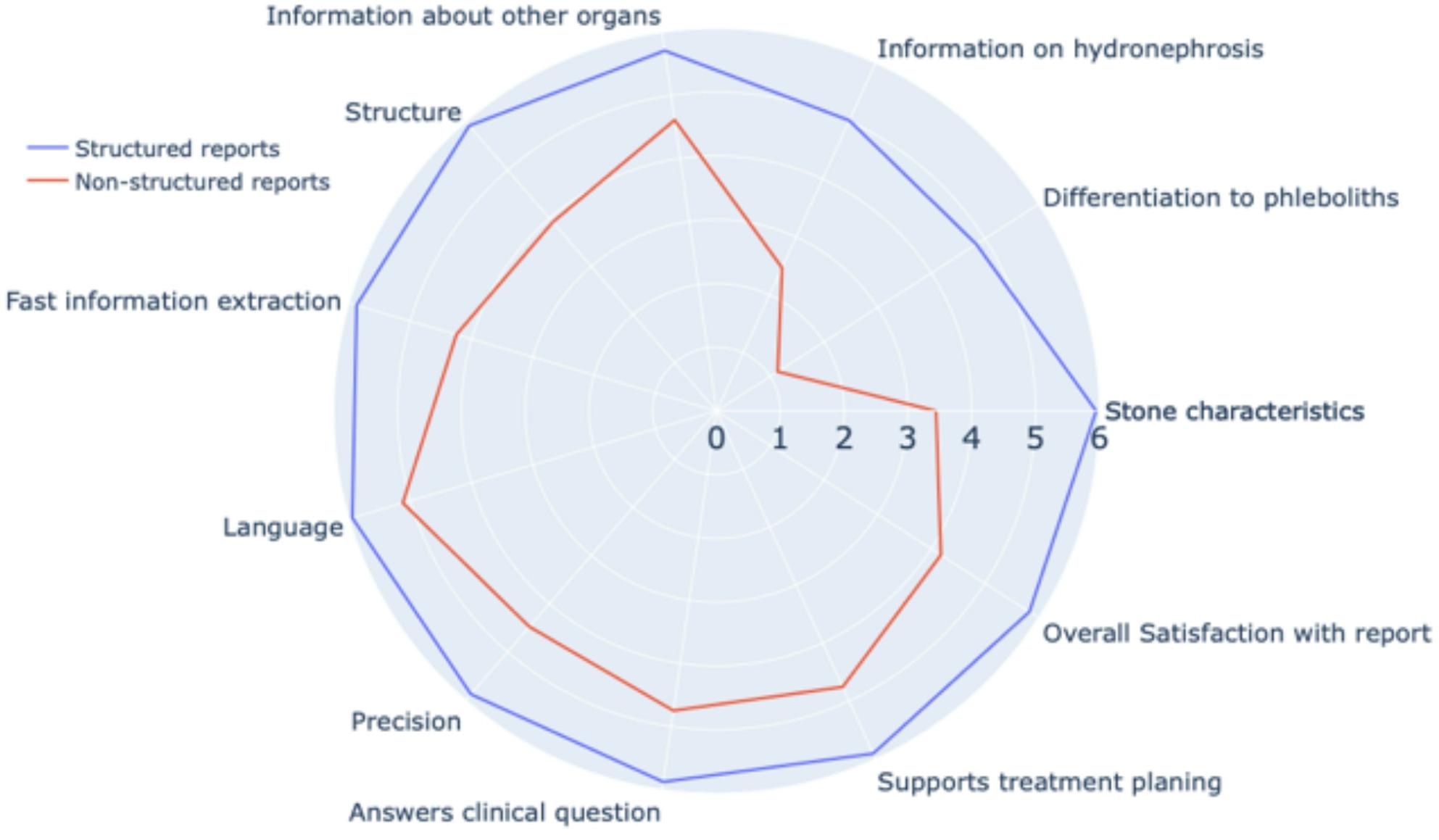
Spider Chart comparing evaluation scores of structured and non-structured reports. Spider chart to visualise the evaluation of questionnaire domains for structured and non-structured reports. Structured reports received better scores in all domains

We observed the biggest differences in scoring of the domain “content” (SR 5.37 ± 1.28 vs. NR 2.92 ± 1.45, *p* < 0.001). This included the specification of stone characteristics (location, size, Hounsfield units, dual energy stone analysis), information on specific radiographic signs to distinguish stones and phleboliths, or additional signs of stone disease such as hydronephrosis, perinephritic inflammation/stranding or forniceal rupture). In addition, the domains “formal aspects/comprehensibility” (clear structure, ease of obtaining information, clear language, and precision) (SR 5.9 ± 0.31 vs. NR 4.44 ± 1.18, *p* < 0.001) and “clinical consequences” (the report clearly answers the question and supports treatment planning) yielded significant better results in SR than NR (SR 5.89 ± 0.33 vs. NR 4.75 ± 1.19, *p* < 0.001). Finally, overall satisfaction was significantly higher in SR than NR (SR 5.83 ± 0.56 vs. NR 4.18 ± 1.13, *p* < 0.001) (Fig. 2, Supplementary Table S2). All reports were completed according to the assigned format without protocol deviations (Supplementary Report S3).

## Discussion

The need for standardised reporting has been advocated by many radiological societies as evident in numerous statements and publications [1, 2]. The widespread use of corresponding quality-tested templates requires several prerequisites: (1) Templates must be developed based on established processes to ensure their quality and acceptance [4]. (2) Templates must be easy to use. Dictating a report may be easier and faster than filling in a complex digital template. It seems essential that templates be integrated into available imaging systems employing an interoperable format [6, 7]. (3) Templates must have recognisable additional benefits for those writing a report – i.e., the radiologist, and those who read the reports such the treating physician or a medical scientist.

Our group has developed and published a template for the standardised, structured reporting of non-contrast computed tomography for urinary stones [3]. This was initiated as a subproject for the *Nationwide Registry for RECurrent URolithiasis in the Upper Urinary Tract* - RECUR [8]. Our main objective was to establish SR of CT scans to be able to add these findings to our fully automated registry. Based on individual numeric values and selected items from drop down menus, SR will enable the automated integration of radiological findings into databases. SR will also facilitate extraction of information for scientific evaluations and the use of data mining techniques (for secondary data use) [9].

However, from the clinician’s point of view, the most important quality characteristics are the accuracy and completeness of radiology reports to enable informed clinical decision-making. In the current study, we demonstrated that SR led to greater satisfaction among urologists at all training levels as compared to free-text reports. This proved to be true in terms of both content and formal aspects. The template-based reports proved superior to free-text reports in their completeness, in avoiding redundancies or ambiguities, and in their clinical applicability. Our findings concur with those described in other medical fields and across all imaging modalities, including computed tomography (CT), magnetic resonance imaging (MRI), and chest radiography. A study by Gassenmaier et al. on MRI shoulder imaging indicated that structured reports facilitate easier comprehension and expedite the extraction of relevant information [10]. In the context of multiple sclerosis, research by Alessandrino et al. suggested that structured MRI reports convey more comprehensive information [11]. A study on CT reports on acute pulmonary embolism by Sabel et al. revealed better content, clinical utility, and clarity with structured formats [12]. Furthermore, Marovici et al.‘s study underscored the effectiveness and completeness of structured reports in chest radiography [13]. As have others, our study confirmed that SR was helpful in both answering the clinical question and in planning treatment [14]. Moreover, SR employs a standardised formal language enabling fast and effective information extraction as well as the establishment of a shared vocabulary [15]. This may help to avoid ambiguities between specialists from different medical fields and improve the overall workflow.

A key advantage of the structured reporting template lies in its ability to standardize the inclusion of radiological features that are often inconsistently reported in free-text formats. In particular, the clear differentiation between urinary stones and phleboliths is of substantial clinical importance. Misinterpretation of phleboliths as ureteral calculi can lead to unnecessary diagnostic procedures or even inappropriate interventions [16]. Structured reporting ensures the documentation of specific radiographic signs—such as the soft tissue rim sign or comet tail sign—that support this differentiation and thus enhance diagnostic accuracy [17]. Similarly, the inclusion of secondary signs of obstructive uropathy, such as hydronephrosis, perinephritic stranding, or signs of fornix rupture, provides critical context for urologists in assessing the clinical relevance of a detected stone. These findings are essential for determining urgency and planning interventions [18]. By ensuring these features are systematically included, structured reporting directly supports more informed and timely clinical decision-making.

It is obvious that the widespread use of templates depends on the radiologists’ willingness to implement them in daily practice. Templates force radiologists to comply with a structured framework that reduces their personal freedom of expression. They also mandate a more detailed description of symptoms and abnormalities potentially considered unnecessary and time consuming. On the other hand, they can complicate the description of rare findings within specific categories. However, leading radiologic societies agree that the further development and implementation of standardised reporting is a major goal within the next decade [1, 2].

Our study is limited by its monocentric design and the exclusive use of reports written in German. It was not possible to blind the radiologists regarding the report format, and due to structural differences, urologists were likely able to identify SR and NR reports during evaluation, which could have introduced a bias in favor of SR. Furthermore, the number of evaluating urologists was limited to five individuals from a single institution, which may affect the generalizability of our results. Differences in clinical routines, language, and familiarity with structured reporting in other institutions or countries may influence how SR is perceived and valued. Additionally, we did not assess the time efficiency of the reporting process or the satisfaction of the radiologists creating the reports—both of which are important factors for the practical implementation and long-term sustainability of structured reporting systems. The questionnaire used in this study was translated into German and adapted to the context of urinary stone diagnostics using a committee-based approach among the study authors. While this ensured contextual and clinical relevance, no formal forward-backward translation was performed.

Therefore, future studies should aim to include multilingual and multicenter settings with a broader range of evaluators, as well as a focus on workflow integration, radiologist experience, and validated tools, to confirm and expand upon our findings and support the international dissemination of the template.

## Conclusion

Urologists rated the standardised, structured reporting of non-contrast computed tomography for urinary stones as being superior to free text reporting in both content and formal aspects. The superiority of SR in terms of completeness, avoiding redundancies or ambiguities, and clarity supports urologists in both clinical decision-making and treatment planning. In addition, SR supports the use of AI technologies to extract and analyse data for scientific evaluations. We plan to continue to disseminate the suggested template by including translated and validated versions.

## Supplementary Information

Supplementary Material 1.

## Data Availability

The dataset, trial protocol and statistical analysis plan generated are not publicly available but can be obtained from the corresponding author upon reasonable request.

## Abbreviations

AI: Artificial intelligence
CT: Computed tomography
DGU: Deutsche gesellschaft für urologie (German Society of Urology)
DRG: Deutsche röntgengesellschaft (German Radiologic Society)
MRI: Magnetic resonance imaging
NCCT: Non–contrast enhanced computed tomography
NR: Non–structured reports
SR: Structured reports
