# Supplementary material for: Structured template for standardised reporting of non-contrast computed tomography in urinary stone disease as a superior tool for urologists – a randomised controlled trial
Source: BMC Urol. 2026 Apr 6;26:94. doi: 10.1186/s12894-026-02134-0 (PMC13081251; doi:10.1186/s12894-026-02134-0)
Supplement: Supplementary file 1 — Supplementary Material 1. [file 12894_2026_2134_MOESM1_ESM.docx]

**Structured template for standardised reporting of non-contrast computed tomography in urinary stone disease as a superior tool for urologists – A randomised controlled trial**

**Supplementary Table S1 – Questionnaire (German/English)**

| 6 = Stimme voll und ganz zu  5 = Stimme zu  4 = Stimme vorwiegend zu  3 = Stimme nur teilweise zu  2 = Stimme nicht zu  1 = Stimme überhaupt nicht zu  X = Diese Angaben sind nicht oder nur teilweise vorhanden, aber aus meiner Sicht auch nicht notwendig | | | | | | | |
| --- | --- | --- | --- | --- | --- | --- | --- |
| Content | 6 | 5 | 4 | 3 | 2 | 1 | X |
| Die wichtigsten Steincharakteristika (Lage, max. Durchmesser, Volumen, mittlere Dichte, Substanz nach Dual-Energy-Analyse) sind gut beschrieben |  |  |  |  |  |  |  |
| *Nur bei Harnleiterstein:*  Zusätzliche radiographische Angaben, die eine Unterscheidung zu Phlebolithen ermöglichen sind beschrieben (Soft tissue rim sign, zentrale Dichteabsenkung, Comet tail sign)  Zusätzliche Angaben zu Harntransportstörungen und indirekte Zeichen einer Stein-bedingten Inflammation oder Fornixruptur sind vorhanden (perirenales bzw. periureterales Ödem) |  |  |  |  |  |  |  |
|  |  |  |  |  |  |  |  |
| Die Befundung anderer Organe ist ausreichend und gut dargestellt |  |  |  |  |  |  |  |
| Formal aspects/ comprehensibility | | | | | | | |
| Der Befund ist gut strukturiert |  |  |  |  |  |  |  |
| Die nötigen Informationen lassen sich schnell aus dem Befund herauslesen |  |  |  |  |  |  |  |
| Die Sprache des Berichts ist eindeutig und leicht zu verstehen |  |  |  |  |  |  |  |
| Der Befund ist präzise und enthält keine unnötigen Informationen |  |  |  |  |  |  |  |
| Clinical consequences | | | | | | | |
| Der Bericht beantwortet die klinische Fragstellung |  |  |  |  |  |  |  |
| Anhand des Berichts lässt sich eine eindeutige Therapie ableiten |  |  |  |  |  |  |  |
| Overall report quality | | | | | | | |
| Ich bin mit dem radiologischen Befund zufrieden |  |  |  |  |  |  |  |

| 6 = Strongly Agree  5 = Agree  4 = Slightly Agree  3 = Partly Agree  2 = Disagree  1 = Strongly Diagree  X = This information is not available or only partially available, but in my opinion it is not necessary | | | | | | | |
| --- | --- | --- | --- | --- | --- | --- | --- |
| Content | 6 | 5 | 4 | 3 | 2 | 1 | X |
| The most important stone characteristics (location, maximum diameter, volume, average density, dual energy analysis) are well described. |  |  |  |  |  |  |  |
| Only for ureteral stones:  Additional radiographic features are described enabling differentiation from phleboliths (soft tissue rim sign, central density reduction, comet tail sign)  Additional information on hydronephrosis and indirect signs of stone-related inflammation or fornix rupture are present (perirenal or periureteral oedema) |  |  |  |  |  |  |  |
|  |  |  |  |  |  |  |  |
| Other organs’ findings are sufficient and well presented. |  |  |  |  |  |  |  |
| Formal aspects/ comprehensibility | | | | | | | |
| The report is clearly structured. |  |  |  |  |  |  |  |
| Relevant information is easy to access. |  |  |  |  |  |  |  |
| The language in the report is clear and easy to understand. |  |  |  |  |  |  |  |
| The report is concise and contains no unnecessary information. |  |  |  |  |  |  |  |
| Clinical consequences | | | | | | | |
| The report answers my clinical question. |  |  |  |  |  |  |  |
| A decision on further clinical management can be made based on the report. |  |  |  |  |  |  |  |
| Overall report quality | | | | | | | |
| I am satisfied with the radiological report. |  |  |  |  |  |  |  |

**Questionnaire.** The questionnaire in German and English, used by urologists to evaluate radiological reports across various categories.

**Supplementary Table S2 – Results**

|  | SR | SD | NR | SD | p |
| --- | --- | --- | --- | --- | --- |
| **Total** | 5.66 | 0.72 | 3.89 | 1.28 | <0.01 |
| **Content** | 5.37 | 1.28 | 2.92 | 1.45 | <0.01 |
| Stone characteristics | 5.95 | 0.27 | 3.44 | 1.06 | <0.01 |
| Differentiation to phleboliths | 4.84 | 2.1 | 1.14 | 1.23 | <0.01 |
| Information on hydronephrosis | 5.01 | 2.1 | 2.47 | 1.98 | <0.01 |
| Information about other organs | 5.71 | 0.55 | 4.61 | 1.6 | <0.01 |
| **Formal aspects** | 5.9 | 0.31 | 4.44 | 1.18 | <0.01 |
| Structure | 5.92 | 0.38 | 3.92 | 1.24 | <0.01 |
| Rapid information extraction | 5.88 | 0.31 | 4.25 | 1.21 | <0.01 |
| Language | 5.95 | 0.21 | 5.12 | 1.15 | <0.01 |
| Precision | 5.88 | 0.35 | 4.48 | 1.17 | <0.01 |
| Clinical consequences | 5.89 | 0.33 | 4.75 | 1.19 | <0.01 |
| Answers clinical question | 5.88 | 0.35 | 4.75 | 1.16 | <0.01 |
| Therapeutic consequence | 5.91 | 0.32 | 4.76 | 1.22 | <0.01 |
| Report quality/Satisfaction | 5.83 | 0.56 | 4.18 | 1.13 | <0.01 |

**Result of questionnaires.** SR = Structured reports, NR = non-structured reports. SD = Standard Deviation.

**Supplementary Report – S3**

#### Structured Report (SR) - German


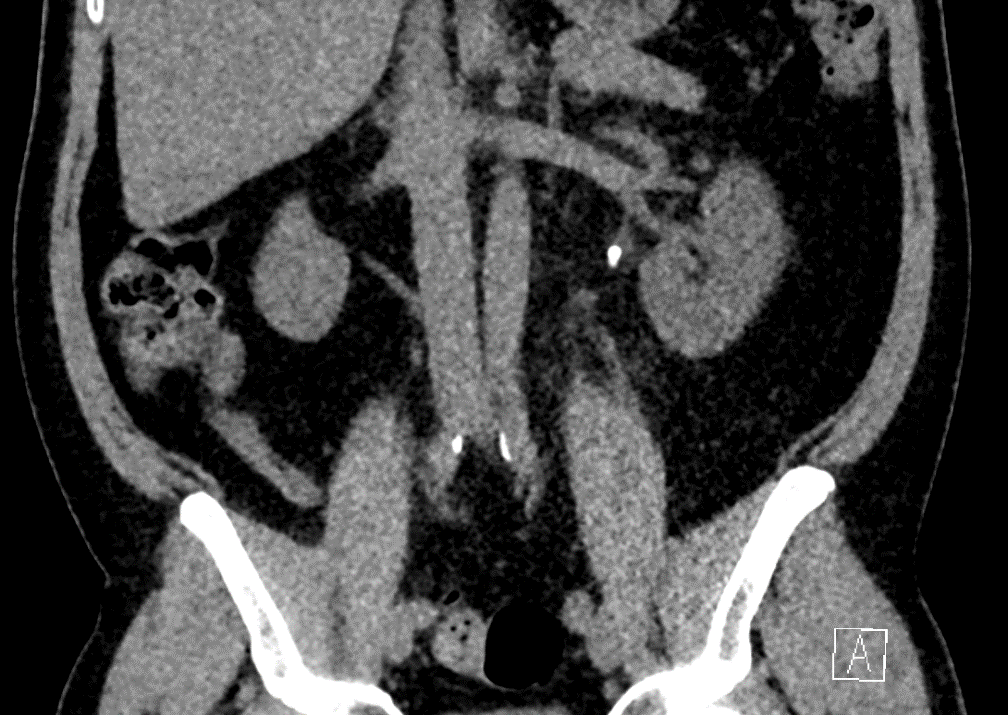


Urolithiasis: Konkrement

Lage: Proximales Ureterdrittel links, zuvor untere Kelchgruppe

Maximaler Durchmesser: 7 x 6 mm

Volumen: 224 mm³

Mittlere Dichte: 1088 HU

Substanz nach Dual-Energy Analyse: Hydroxylapatit

Soft tissue rim sign: negativ

Zentrale Dichteabsenkung: Nicht vorhanden

Comet tail sign: Negativ

Weitere Konkremente: Keine

Harntransportstörung:

Aufweitung des Nierenbeckenkelchsystems:

Links: Grad II

Rechts: Keine

Aufweitung des Ureters:

Links: Bis auf Höhe des proximalen Drittels, max. Durchmesser: 10 mm

Rechts: Keine

Perirenales Ödem: links

Periureterales Ödem:

Links: im proximalen Drittel

Rechts: keines

Bauchorgane: Unauffällig

Gefäße: Unauffällig

Lymphknoten: Unauffällig

Skelett: Unauffällig

Basaler Thorax: Unauffällig

Beurteilung

Hydroxylapatit-Konkrement im proximalen Ureter links (zuvor untere Kelchgruppe) mit 7 mm Maximaldurchmesser und vorgeschalteter Harntransportstörung des rechten Nierenbeckenkelchsystems und Harnleiters Grad 2.

**Structured Report (SR) – English**

Urolithiasis: Calculus
Location: Proximal third of the left ureter, previously in the lower pole calyx
Maximal dimensions: 7 × 6 mm
Volume: 224 mm³
Mean attenuation: 1088 HU
Composition by dual-energy analysis: Hydroxyapatite
Soft-tissue rim sign: Negative
Central low attenuation: Absent
Comet-tail sign: Negative
Additional calculi: None

Urinary tract obstruction:

Pelvicalyceal dilatation (hydronephrosis):

Left: Grade II

Right: None

Ureteral dilatation (hydroureter):

Left: Up to the level of the proximal third; maximal diameter 10 mm

Right: None

Perirenal edema: Left

Periureteral edema/stranding:

Left: In the proximal third

Right: None

Abdominal organs: Unremarkable
Vasculature: Unremarkable
Lymph nodes: No lymphadenopathy
Skeleton: Unremarkable
Visualized lung bases: Unremarkable

Summary: Hydroxyapatite calculus in the proximal left ureter (previously in the lower pole calyx) measuring up to 7 mm, with upstream grade 2 obstruction of the left pelvicalyceal system and ureter.

**Unstructured Report - German**


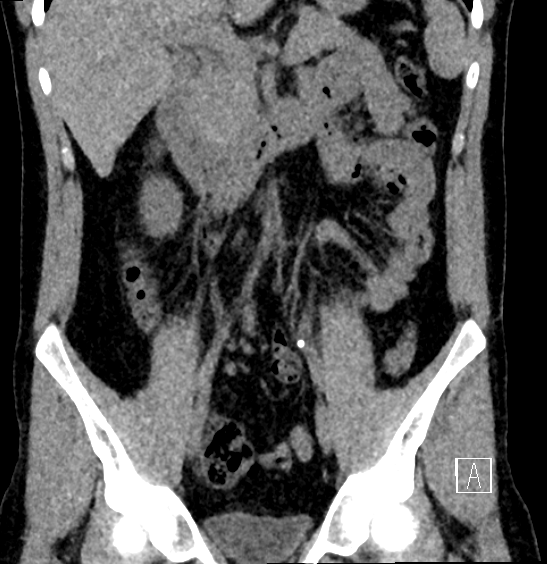


4 x 4 x 3 mm messendes Konkrement im mittleren Ureter linksseitig mit weiter proximal geringgradiger langstreckiger Erweiterung des Ureters. Kein signifikanter Harnaufstau. Weiteres kleines Konkrement in der oberen Kelchgruppe links mit ca. 1-2 mm (2/14). Kein Nachweis eines intravesikalen Konkrements. Rechts kein Harnkonkrement. Rechts kein Harnaufstau. Kein freies intraabdominelles Gas. Keine freie intraabdominelle Flüssigkeit. Keine Ileuszeichen. Unauffällige Kontur der Bauchorgane soweit beurteilbar. Keine malignomsuspekten ossären Läsionen.

Beurteilung

4 x 4 x 3 mm messendes Konkrement im mittleren Ureterdrittel linksseitig (Höhe LWK 5/SWK 1), in der DE-Auswertung Oxalat-haltig. Konsekutiv geringe Erweiterung des Ureters proximal des Konkrementes.

#### Unstructured Report – English

4 × 4 × 3 mm calculus in the mid left ureter with mild, long-segment proximal ureteral dilatation. No significant hydronephrosis. Additional small calculus in the left upper-pole calyx measuring approximately 1–2 mm (series 2, image 14). No intravesical calculus. Right side: no urinary calculus; no hydronephrosis. No free intra-abdominal air or fluid. No radiologic signs of bowel obstruction. Contours of the abdominal organs unremarkable where assessable. No osseous lesions suspicious for malignancy.

**Summary:** 4 × 4 × 3 mm calculus in the mid third of the left ureter (at the L5/S1 level); dual-energy evaluation indicates an oxalate-containing composition. Mild upstream dilatation of the ureter proximal to the calculus.
